# Growth, maturity status and performance of youth soccer players by playing status as adults

**DOI:** 10.5114/biolsport.2026.156225

**Published:** 2026-01-02

**Authors:** Robert M. Malina, Luís Horta, João P. Duarte, Nuno Silva-Borges, Tomas Oliveira, Jorge M. Celis-Moreno, Oscar M. Tavares, Andre Seabra, Jan M. Konarski, Manuel J. Coelho-e-Silva

**Affiliations:** 1 University of Texas at Austin, Department of Kinesiology and Health Education, Austin, Texas, United States of America; 2 University of Louisville, School of Public Health and Information Sciences, Louisville, Kentucky, United States of America; 3 Centro Hospitalar Universitário de Lisboa Central (CHULC), Centro Clínico Académico de Lisboa (CCAL), Lisboa, Portugal; 4 University of Coimbra, FCDEF, Coimbra, Portugal; 5 State University of Londrina, Physical Education and Sport Center (GEPEMENE), Londrina, Paraná, Brazil; 6 University of Santo Tomas, Bogotá, Colombia; 7 Polytechnic Institute of Coimbra, Coimbra Health School, Coimbra, Portugal; 8 Portugal Football School, Portuguese Football Federation, Lisbon Portugal; 9 Poznań University of Physical Education, Sports Science, Poznań, Poland; 10 University of Coimbra, CIPER, Coimbra, Portugal

**Keywords:** Youth athletes, Talent development, Skeletal age, Pubertal status, Coach perceptions

## Abstract

To evaluate the growth, maturity and performance characteristics of select youth soccer players grouped by subsequent playing status as adults. Height, weight, skeletal and pubertal maturity status, and performances on jumping, speed and aerobic endurance tasks were measured in 1998–1999 for 135 players 11–12 (n=29), 14–15 (n=61) and 16–17 (n=45) years. Based on records of the Portuguese Soccer Federation for the 2018–2019 season, 63 of the players were not involved in soccer as adults, while 47 played at the amateur level and 25 played professionally. Among players 11–12 years, those playing professionally as adults performed significantly better in the jump and sprint compared to peers no longer playing, while among players 14–15 years, those playing professionally as adults were significantly taller and performed better in the sprint as youth. Among players 16–17 years, in contrast, those playing soccer as an amateur or professionally and those no longer involved as adults did not differ as youth in body size, skeletal and pubertal maturity status, and performances. Comparisons of the youth characteristics of those playing soccer as adults and those no longer involved indicated no consistent pattern in the three competitive age groups considered. Unfortunately, information on soccer-specific skills and coach perceptions of skills and characteristics of youth players was not available.

## INTRODUCTION

The growth and maturity status of male youth soccer players 10 through 18 years is reasonably well documented [1, 2]. Heights and weights of youth players have increased, on average, across studies spanning the late 1970s through 2015 [3], while the skeletal maturity status of soccer players 10 through 16 years suggests average maturity status in late childhood and early adolescence, but systematic selection of players advanced in maturity status beginning at about 12–13 years [4]. Studies of the functional characteristics of youth players highlight interactions among CA, body size and maturity status (skeletal, pubertal) as a source of variation in performances on tests of speed, power, agility and endurance [5, 6]. Corresponding data for maturity timing as reflected in estimated age at PHV based on longitudinal samples of European soccer players are not extensive. Studies spanning the 1990s through the present suggest negligible changes in ages at PHV, although the available data suggest slightly earlier ages at PHV in samples of players from southern compared to players in northern and northwestern Europe [7].

Allowing for the trends suggested in studies of the growth, maturity status and performances of youth soccer players, the characteristics of youth players who persist in soccer through adolescence into adulthood compared to peers no longer involved in the sport merit attention. Available data, though limited, indicate variable results among studies addressing the characteristics of adolescent soccer players who persist in the sport into adulthood in France [8, 9], Portugal [10], Germany [11, 12] and Serbia [13].

Given the limited data and somewhat variable results, the present study compares the growth and biological maturity status, and the functional characteristics of youth soccer players 11–12, 14–15 and 16–17 years from several elite clubs in Portugal grouped by their playing status in adulthood, specifically those who persisted in the sport into adulthood at the local and national levels relative to peers who no longer participated in the sport as adults. The groups approximate three phases of adolescence, respectively, early adolescence (11–12 years), the interval immediately after the peak of the growth spurt (14–15 years), and late adolescence (16–17 years). Note that mean ages at peak height velocity for youth soccer players in Europe vary with one exception between 13.4 and 14.2 years [14]. It was thus hypothesized that variation in the growth, maturity status and functional characteristics of youth soccer players who persist in the sport into adulthood would emerge after the interval of the adolescent growth spurt.

## MATERIALS AND METHODS

### Participants

Data for the present study were part of the project “*Prediction factors of sports performance in youth soccer players*”, which followed the guidelines established by the declaration of Helsinki [15]. Formal approval was obtained from the University of Porto [DA.SPGA.2608661], and included agreements with the written consent obtained from parents or legal guardians of the players who were informed that participation was voluntary and that they could withdraw from the study at any time. The sample included 142 youth soccer players 11–17 years from several clubs in Portugal selected for nationally representative youth teams in 1997–1998 [16].

### Growth and Maturity Status

In addition to chronological age (CA), height and weight were measured, hand-wrist radiographs for the assessment of skeletal age (SA) were taken and pubertal status was assessed. The radiographs were assessed with the Fels method [17] by a single, experienced observer (RMM) to estimate SA at the time of observation. The Fels method is based on youth from largely middle class families in southcentral Ohio in the United States. The method requires the evaluation of the maturity status of the carpal bones and the epiphyses and corresponding diaphyses of 13 long bones – the radius, ulna and the metacarpals and phalanges of the first, third and fifth digits. Grades are assigned to the indicators for each bone relative to the described criteria. The presence or absence of the adductor sesamoid of the first metacarpal is also noted and the widths of the epiphysis and metaphysis of each of the long bones are measured. The grades assigned for specific bones and the epiphyseal and metaphyseal widths are entered into a computer program that calculates the SA and its standard error of estimate for the individual. The standard error provides an indication of the error inherent in the assessment. Other methods of assessing skeletal maturation do not provide an estimate of error associated with the assessment. Radiographs of a random sample of 15 players 11.7 to 16.1 years of age were reassessed six months after the initial assessments. The mean difference between SA assessments was 0.08 ± 0.17 year, while the correlation between assessments was 0.99.

Based on the difference between SA minus CA, each player was classified as average, advanced or late in skeletal maturity status. An SA within ± 1.0 year of CA was classified as average or on time; an SA in advance of CA by > 1.0 year was classified as early or advanced, while an SA less than CA by more > 1.0 year was classified as late or delayed [18]. Finally, Pubertal status based on the development of pubic hair and the genitalia was assessed relative to the criteria described by Tanner [19]. The criteria for each characteristic include five stages, with stage 1 indicating the prepubertal state, stages 2, 3 and 4 indicating progress in pubertal status, and stage 5 indicating the mature state [20].

### Functional Characteristics

Three commonly used indicators of functional status were adopted: lower-limb power, speed/agility, and aerobic endurance. Lower-limb power was assessed with the standing long jump measured on a flat surface as the distance (nearest cm) from the take-off line to the point where the heels touch the ground. The best of three trials was retained for analysis. Speed was estimated with a slalom sprint in which the player ran at maximum speed for a distance of 34.2 meters with three changes of direction [21]. The time to cover the distance was recorded with a digital chronometer connected to photoelectric cells (Globus Ergo Timer Timing System, Codogné, Italy). Three trials were given with a 25-s recovery period between trials, and performance was expressed as the mean time of the three trials. Finally, aerobic endurance was estimated with the PACER 20-m multi-stage continuous shuttle endurance test [22] which required the player to run as many 20-m shuttles as possible; the score was the total distance covered (meters) until the athlete was no longer able to maintain the required pace (8.0 km · h^−1^ with increments every minute). A single trial was given.

### Playing Status in Adulthood

The playing status of the baseline sample in 2018–2019 was checked by name and birthdate on publicly available information of the Portuguese Soccer Federation website: https://www.fpf.pt/pt/Jogadores/Pesquisar-JogadoresInternacionais/NationalTeamId/1619469.

The available information indicated the careers of individual soccer players including specific clubs on a yearly basis.

Among the 142 players, 70 (49%) were no longer involved in soccer as adults, while 47 (33%) played in amateur level clubs (46 of the 47 of the clubs were in Portugal) and 25 (18%) signed professional contracts nationally or internationally. Among the latter 7 played on the Portuguese senior national team or in the Champions League. The baseline sample of 142 players assessed in 1998–1999, included amateur and professional players who at follow-up were represented in all CA groups except 13 years; the latter included six players and none was involved in soccer as an adult. In addition, data for one player 14 years of age who was no longer involved in soccer was incomplete. The remaining sample of 135 players was thus partitioned into three competitive CA groups for analysis: 11–12 years (n = 29), 14–15 years (n = 61) and 16–17 years (n = 45).

### Analysis

Descriptive statistics (means, standard deviations, medians, and ranges) were computed for years of training in soccer and weekly training volume at the time of data collection. The baseline sample included youth players assessed in 1997–1998 who were grouped into three competitive age categories (11–12, 14–15, and 16–17 years). The career follow-up was individually checked in 2018–2019 to determine adult playing status: no longer involved in soccer (NLP), amateur (A), or professional (P). One-way analysis of variance (ANOVA) was used to examine the effect of adult playing status (NLP, A, P) separately within each age group. Effect size estimates based on eta-squared (η^2^) were interpreted as follows [23]: trivial (η^2^ < 0.01), small (0.01 ≤ η^2^ < 0.06), moderate (0.06 ≤ η^2^ < 0.14), and large (η^2^ ≥ 0.14). When the F-test was statistically significant, Bonferroni-adjusted post hoc comparisons were conducted to identify specific pairwise differences. In view of the small sample sizes in some subgroups, a less stringent significance level (p ≤ 0.10) was adopted and explicitly reported, to highlight potentially meaningful trends. In all cases, statistical interpretation was complemented by Cohen’s d effect size estimates for pairwise comparisons, categorized as follows [24]: trivial (d < 0.2), small (0.2 ≤ d < 0.6), moderate (0.6 ≤ d < 1.2), large (1.2 ≤ d < 2.0), very large (2.0 ≤ d < 4.0), and nearly perfect (d ≥ 4.0). Finally, associations between maturity status classifications (skeletal maturity status, pubic hair stage, and genital stage) and adult playing status were assessed within each of the three age groups using chisquare (χ^2^) tests with likelihood ratio statistics. All statistical analyses were performed using SPSS (IBM SPSS Statistics, Version 28, Armonk, NY, USA).

## RESULTS

Descriptive statistics for training experience and weekly training volume during adolescence are presented in Table 1. As expected, players in older CA groups accumulated more years of soccer training and engaged in greater weekly training volumes. Among players 11–12 years at baseline, those playing professionally as adults tended to have, on average, slightly more years of training (4.0 ± 0.0 years) compared to their amateur peers (3.8 ± 0.4 years) and those no-longer-playing (3.4 ± 0.5 years) peers. A similar trend was noted among players 14–15 years, but the differences were more marked between those playing professionally and as amateurs (respectively, 5.9 ± 2.0 years and 4.9 ± 1.9 years); those no longer player had slightly fewer years of training (4.5 ± 1.3) years. Among players 16–17 years, those playing professionally and as amateurs differed negligibly in years of training (respectively, 6.3 ± 0.8 years and 6.2 ± 1.3 years), while those no longer playing had fewer years of training (5.2 ± 1.3 years). Of interest, hours of training per week as youth in each of the three CA groups was, on average, slightly higher among those no long playing soccer compared to their amateur and professional peers.

**TABLE 1:** Sample sizes, years of training and hours of training per week (h/wk) in 1997–1998 among youth players in three competitive age groups classified by playing status as adults in 2018–2019.

Competitive Age Groups	Playing Status in adulthood											

	No Longer Playing				Amateur				Professional			

	M	SD	Md	R	M	SD	Md	R	M	SD	Md	R
**11–12 yrs (n = 29)**	**n = 16**				**n = 6**				**n = 7**			
Years training, yrs	3.4	0.5	3	(3–4)	3.8	0.4	4	(3–4)	4.0	0.0	4	(4)
Training, h/wk	5.4	0.7	6	(4–6)	4.7	0.6	5	(4–6)	4.0	0.0	4	(4)

**14–15 yrs (n = 61)**	**n = 32**				**n = 22**				**n = 7**			
Years training, yrs	4.5	1.3	4	(2–8)	4.9	1.9	5	(2–9)	5.9	2.0	7	(2–8)
Training, h/wk	6.2	1.3	6	(4–8)	6.1	1.7	6	(4–10)	5.9	1.9	6	(5–8)

**16–17 yrs (n = 65)**	**n = 15**				**n = 19**				**n = 11**			
Years training, yrs	5.2	1.3	5	(3–8)	6.2	1.3	6	(3–8)	6.3	0.8	6	(4–8)
Training, h/wk	8.4	1.3	8	(6–10)	7.8	1.0	8	(6–10)	8.1	1.4	8	(6–10)

*Means (M), standard deviations (SD), medians and (R) ranges.

Table 2 presents results of the one-way ANOVAs comparing CA, SA, anthropometric and physical performance variables of the players as youth grouped by adult playing status within each baseline CA group. Among players 11–12 years, CA, SA, the SA-CA difference, height, weight and endurance performance did not differ significantly among the three groups. However, professional and amateur players performed significantly better in standing long jump (F = 4.78, p = 0.02, η^2^ = 0.27) and sprint speed (F = 6.66, p < 0.01, η^2^ = 0.34) compared to those no longer involved in the sport. Pairwise comparisons indicated a large effect in the standing long jump as youth between those playing professionally and no longer playing (d = 1.21), and between amateur players and those no longer playing (d = 0.93). For sprint performance, large effects were also observed between professionals and no-longer-playing peers (d = 1.44), and between amateurs and those no longer playing (d = 1.20).

**TABLE 2:** Chronological age (CA), body size, skeletal age (SA) and performances of soccer players 11–12, 14–15 and 16–17 years of age in 1997–1998 grouped by playing status in soccer as adults in 2018–2019: means ± standard deviations.

Age groups	Characteristics in 1997–1998	Adult playing status (2018–2019)			F	p	η^2^				
								Effect size (Cohen d-value)			observation
	
		NLP	Amateur	Professional				NLP vs A	NLP vs P	A vs. P	
**11–12 yrs**	n = 29	n = 16	n = 6	n = 7							
	CA, yrs	12.35 ± 0.48	12.19 ± 0.67	12.24 ± 0.46	0.25	0.78	0.02				
	Height, cm	150.1 ± 10.1	152.8 ± 5.7	152.1 ± 6.4	0.28	0.76	0.02				
	Weight, kg	42.4 ± 8.5	44.1 ± 5.9	42.2 ± 6.8	0.13	0.88	0.01				
	Fels SA, yrs	12.34 ± 1.59	12.82 ± 1.17	12.04 ± 1.41	0.45	0.64	0.03				
	SA–CA, yrs	–0.01 ± 1.47	0.63 ± 0.55	–0.20 ± 1.33	0.73	0.49	0.05				
	Jump, cm	186 ± 24	210 ± 27	212 ± 11	4.78	0.02	0.27	0.96^*^	1.30^**^		(A = P > NLP)
	Speed, sec	8.18 ± 0.65	7.54 ± 0.42	7.40 ± 0.17	6.66	< 0.01	0.34	1.13^*^	1.63^***^		(A = P > NLP)
	Endurance, m	2488 ± 684	2080 ± 462	2411 ± 482	1.00	0.38	0.07				

**14–15 yrs**	n = 61	n = 32	n = 22	n = 7							
	CA, yrs	15.05 ± 0.61	15.46 ± 0.46	15.35 ± 0.45	3.77	0.03	0.11	0.74^**^			(A > NLP)
	Height, cm	171.4 ± 5.2	174.3 ± 4.9	179.0 ± 1.3	7.62	< 0.01	0.21		1.85^***^	1.22^*^	(P > A = NLP)
	Weight, kg	64.4 ± 6.9	67.1 ± 6.5	68.5 ± 7.1	1.61	0.21	0.05				
	^†^CA, yrs	15.01 ± 0.61	15.42 ± 0.47	15.35 ± 0.45	3.72	0.03	0.12	0.75^**^			(A > NLP)
	^†^Fels SA, yrs	15.69 ± 1.44	16.27 ± 1.17	15.82 ± 1.20	1.14	0.33	0.04				
	^†^SA–CA, yrs	0.68 ± 1.04	0.84 ± 1.25	0.48 ± 1.03	0.30	0.74	0.01				
	Jump, cm	224 ± 18	234 ± 16	241 ± 24	3.22	0.05	0.10		0.79^*^		(P > NLP)
	Speed, sec	7.47 ± 0.58	7.11 ± 0.37	7.06 ± 0.25	4.44	0.02	0.13	0.74^**^			(A > NLP)
	Endurance, m	4096 ± 861	3869 ± 464	3794 ± 496	0.93	0.40	0.03				

**16–17 yrs**	n = 45	n = 15	n = 19	n = 11							
	CA, yrs	16.64 ± 0.53	16.91 ± 0.70	16.95 ± 0.63	0.99	0.38	0.05				
	Height, cm	175.9 ± 5.1	175.3 ± 4.9	174.8 ± 5.6	0.18	0.84	0.01				
	Weight, kg	68.9 ± 7.5	69.8 ± 5.4	70.1 ± 7.0	0.13	0.88	0.01				
	^††^CA, yrs	16.66 ± 0.59	16.79 ± 0.63	16.78 ± 0.67	0.14	0.87	0.01				
	^††^Fels SA, yrs	17.09 ± 0.66	17.28 ± 0.65	16.88 ± 0.56	1.06	0.36	0.06				
	^††^SA–CA, yrs	0.42 ± 0.80	0.49 ± 0.73	0.10 ± 0.68	0.75	0.48	0.05				
	Jump, cm	235 ± 16	240 ± 17	232 ± 15	0.81	0.45	0.04				
	Speed, sec	7.15 ± 0.35	6.95 ± 0.40	7.078 ± 0.43	1.01	0.37	0.05				
	Endurance, m	3971 ± 599	4148 ± 809	4313 ± 618	0.78	0.47	0.04				


NLP (No Longer Playing); A (amateur); P (professional)

Jump (standing long jump); Speed (34.2 m slalom sprint); Endurance (PACER: multi-stage 20 m shuttle run)

† Sample non-skeletally (NLP = 30; A = 20; P = 7)

†† Sample non-skeletally (NLP = 11; A = 15; P = 8)

* (p < 0.10);

** (p < 0.05);

*** (p < 0.01).

Among players 14–15 years, those playing soccer professionally were significantly taller as youth compared to those playing soccer as amateurs and those no longer playing (F = 7.62, p < 0.01, η^2^ = 0.21). The difference in height indicated a large effect between professionals and amateurs (d = 1.14) and between professionals and those no longer playing (d = 1.65). Amateurs had a higher CA and faster sprint times than those no longer playing; the sprint comparison indicated a moderate effect (d = 0.72). Differences in the standing long jump approached significance (p = 0.05), with a moderate effect size (η^2^ = 0.10), but pairwise d values did not exceed the threshold for a large effect. SA and the SA-CA difference did not differ among the three groups.

Among players 16–17 years, in contrast, no statistically significant differences were noted among the three playing status groups for CA, height, weight, SA, SA-CA and the three physical performance tasks. Effect sizes were also small for all comparisons (η^2^ < 0.06), and d values for pairwise comparisons did not indicate meaningful effects.

Table 3 summarizes the distributions of the players by skeletal maturity status and stages of pubic hair and genital development in each of the three CA groups of youth players at baseline by playing status as adults. Chi-square tests for the distribution of skeletal maturity status in each of the three CA groups were not significant. Likelihood ratios ranged from 2.31 to 3.20, indicating weak associations. Distributions of the stages of pubertal development among players in the adult playing groups also did not differ significantly. For example, among players 11–12 years, a larger proportion of those no longer playing soccer were at early pubertal stages (i.e., genital stage 1 or 2), but this trend was not statistically significant (p = 0.26; LR = 10.62). Among players 14–15 years and 16–17 years, the distributions of pubic hair and genital stages were relatively similar across playing among adult playing groups.

**TABLE 3:** Skeletal and pubertal maturity status of the soccer players in the three competitive chronological age groups (11–12 years, 14–15 years, 16–17 years) in 1997–1998 classified by playing status in soccer as adults in 2018–2019.

Age groups	Indicator	group	Adult playing status(2018–2019)			χ^2^	df	p	likeliho-odratio

			NLP	Amateur	Professiona				
**11–12 yrs(n = 29)**	Skeletal	Late	(n = 16) 4	(n = 6) 0	(n = 7) 2	2.31	4	0.69	3.46
		Average	9	4	4				
		Early	3	2	1				

	Pubic Hair stage	1	11	2	4	4.09	6	0.67	4.39
		2	2	2	2				
		3	2	2	1				
		4	1	0	0				

	Genital stage	1	8	0	2	7.77	6	0.26	10.62
		2	5	4	5				
		3	1	1	0				
		4	2	1	0				

**14–15 yrs(n = 61)**	Skeletal	Late	(n = 32) 2	(n = 22) 1	(n = 7) 1	2.71	6	0.84	3.03
		Average	15	8	4				
		Early	13	11	2				
		Mature	2	2	0				

	Pubic Hair stage	2	1	0	0	2.13	4	0.71	2.50
		4	24	14	5				
		5	7	8	2				

	Genital stage	2	1	0	0	2.14	4	0.71	2.44
		4	24	16	4				
		5	7	6	3				

**16–17 yrs(n = 45)**	Skeletal	Late	(n = 15) 1	(n = 19) 0	(n = 11) 0	2.99	6	0.81	3.20
		Average	8	11	7				
		Early	2	4	1				
		Mature	4	4	3				

	Pubic Hair stage	4	8	10	7	0.39	2	0.82	0.39
		5	7	9	4				

	Genital stage	4	10	10	8	1.39	2	0.50	1.39
		5	5	9	3				

NLP (No Longer Playing); df (degree of freedoon); LR (likelihood ratio); p (significance value).

## DISCUSSION

Studies considering the growth and maturity characteristics of youth who persist in soccer into adulthood are relatively limited. Observations at 11–12 years of age for those in their early 30’s (~32–33 yrs) at the time of follow-up in the present study (Table 2) were generally consistent with those for soccer players of the same competitive CA group in the Portuguese midlands who were classified by playing status in their early 20s [25]. In both studies, those playing at amateur (n = 6) and professional (n = 7) levels and those no longer involved in the sport (n = 16) as adults in the present study and those playing nationally (n = 5) and regionally (n = 10) and no longer involved in soccer (n = 72) in their early 20s did not differ, on average, in CA, SA, height and weight, and in the distributions by skeletal maturity and pubertal status. In the present study, those playing professionally did not differ as youth from those playing at the amateur level in the jump and speed/agility tests, but performed better than those no longer involved in soccer; players in the three groups, however, did not differ in the endurance run. In the sample followed-up in their early 20s [25], on the other hand, those playing nationally and locally performed significantly better in the endurance shuttle run (national > regional = no longer playing), but those playing nationally performed significantly better than those no longer involved in the sport on three soccer skills (ball control, dribbling, passing); performances were, on average, also better than those playing regionally, but the difference was significant only for dribbling. In a follow-up study of a national sample of U12 German players at 19–22 years of age [11], there was a consistent gradient in height, weight and performances in a 20 m sprint, agility, dribbling, ball control and shooting during youth by young adult playing status: professionals > semi-professionals > non-professionals.

Among players 14–15 years of age (35–36 years at follow-up), those playing at the amateur level as adults were significantly older chronologically than those no longer involved in soccer, but those playing professionally as adults were significantly taller (Table 2). The three groups, however, did not differ in SA and distributions by skeletal and pubertal status as youth, but those who persisted in soccer into adulthood at the amateur or professional levels performed better on the jumping and speed-agility tasks as youth compared to peers no longer involved in the sport. Players 14–16 years at an elite academy in France who eventually played internationally and professionally in their late teens also did not consistently differ in growth and maturity status compared to their amateur peers, but those playing internationally performed better than amateur peers on jumping and sprinting tests [8]. Of interest, on entry into the academy at 13 years of age, those who eventually played professionally were slightly younger, taller and heavier, and performed slightly better in aerobic endurance, but did not differ in SA and other performance tests compared to peers who did not play professionally [9].

In a study of players 13–14 years in the midlands of Portugal, those who persisted in soccer nationally (n = 5) were chronologically older and taller, but did not differ in skeletal and pubertal maturity status compared to peers playing regionally (n = 15) and peers no longer involved in soccer (n = 52) as young adults (22–25 years); those who persisted in soccer also performed better in the yo-yo endurance run and soccer-specific ball control and passing tests as teens [10]. In the study of U12 German players noted earlier [11], a subsample was followed longitudinally from U12 through U15 playing status [12]; those subsequently classified as elite at 19–22 years of age performed better as youth in agility and three soccer skills, but not in speed, compared to non-elite peers.

In an independent study of 114 players 13–14 years of age from the districts of Aveiro and Coimbra in the midlands of Portugal, 45 field players (i.e., excluding goalkeepers) were selected for the respective regional teams in each district. Although the select players did not differ in CA relative to the 69 non-select peers, the select players were advanced in skeletal maturity status, taller and heavier, and performed better in the squat jump, sprint and two soccer-specific skills – ball control and dribbling [26]. Similarly, select Polish soccer players 13–14 years were advanced in estimated maturity status (percentage of predicted adult height at the time of observation), taller, heavier and stronger, and performed better in a 20 m sprint and the vertical jump compared to non-select peers, while players in the two groups did not differ in the yo-yo endurance shuttle run [27].

In contrast to observations in the present study and in other studies of youth soccer players, a study of 48 elite and sub-elite Serbian soccer players at 22 years of age concluded that proportionally more elite players were classified as late in skeletal maturity status compared to peers at 14 years of age [13]. The conclusion, however, is misleading for two reasons and should be viewed with caution. First, a narrow SA-CA band of ± 0.5 year was used to define maturity groups, and second, assessments of SA were based on the most recent version of the Tanner-Whitehouse radius-ulna-short bone method, TW3 RUS [28]. The narrow band of ± 0.5 year is within the range of error associated with SA assessments, while the TW3 RUS method systematically assigns a lower SA (~1.0 year) for the same maturity score compared to the TW2 RUS version of the method [28, 29]. The criteria for the assessment of each of the 13 bones and associated RUS scores with the TW2 and TW3 protocols do not differ; but SAs assigned to the sum of RUS scores differ for the two versions of the method. Specifically, SAs assigned with TW3 are systematically lower than those assigned with TW2 [29]. Based on the mean RUS score reported for the total sample of 48 Serbian players (CA = 14.5 ± 0.3 years), the mean TW2 RUS SA was 15.5 years and was in advance of the reported TW3 RUS SA of 14.7 years [13]. In an international sample of 194 soccer players 14.5 ± 0.3 years, TW2 RUS and TW3 RUS SAs were, respectively, 15.3 ± 1.2 and 14.2 ± 1.3 years [29]. The contrasting results highlight the need to critically consider variation among methods used to assess skeletal maturity status. In the international sample of non-skeletally mature youth soccer players from which the comparative data at 14 years were extracted, CA specific means for TW3 RUS SAs were, on average, 1.0 to 1.2 years less than TW2 RUS SAs from 11 through 17 years [29].

Observations for players 16–17 years of age who persisted in soccer at the amateur and professional levels did not differ from peers who were no longer involved in soccer in growth and maturity status and the three functional tests (Table 2). Comparative data for late adolescent soccer players are limited. A study of players 16–18 years of age from two Dutch clubs [30] considered the characteristics of 76 players permitted to continue at the respective clubs, labeled as selected, and 37 players not permitted to continue at the respective clubs, labeled as deselected. Players in the respective groups did not differ in CA, height and weight, and in behavioral and psychological characteristics; though not reported, given the CA range, it is likely that most of the players were skeletally mature or approaching skeletal maturity. However, selected players performed better in two of four functional tests (peak and repeated shuttle sprints), two of three technical skills (peak and repeated dribbles) and one of four technical characteristics (positioning and deciding). Moreover, results of a discriminant function analysis indicated three variables which correctly classified 69% of the players as selected or deselected – in order, peak shuttle dribble (functional skill), positioning and deciding (technical characteristic), and peak shuttle sprint (technical skill). Unfortunately, the present study was limited to a jump, slalom sprint and endurance run, and did not consider sport specific technical and behavioral variables.

Although specific data are not available, it has been suggested that the “…mental and technical skills or practice history profiles” of youth players along with their growth, maturity status and fitness skills should be considered in an effort to define the “prerequisites for professional and international soccer…” [8, p. 94]. Of interest, no mention was made of coach perceptions of youth players. In the study of youth players in the midlands of Portugal [10], coaches were asked to evaluate the potential for success of the players at the time of observation (11–12 and 13–14 years); youth who subsequently played soccer nationally as young adults were rated by their coaches at baseline as having significantly higher potential for success compared to peers who subsequently played locally or were no longer involved in the sport. Similarly, a study of Polish soccer players 13–14 years of age noted that select players were rated at baseline by coaches as higher in tactical skills associated with attack and in skills associated with creativity and decision making compared to non-select players [27]. The select and non-select players also differed significantly in overall coach evaluations of potential expressed as the sum of the scores for five elements viewed as essential to performance in soccer.

Although the data are somewhat limited, results of the preceding studies highlight the need for further study of coach perceptions of the abilities and of the potential of youth players, and how they are influenced by or related to the characteristics ordinarily considered in studies of youth players, i.e., size, maturity status, fitness and sport-specific skills. More specifically, what are coaches seeing or observing at these ages that is not ordinarily considered in studies of youth soccer players? This is relevant as expectations of coaches and clubs regarding the potential of youth players, even if subjectively based, influence playing time and may focus attention on specific players which may function to motivate persistence in the sport and influence decisions.

The present study is not without limitations. Data were limited for players 13 years of age at baseline and also at follow-up; this is the CA at which the growth spurt is actively in process. In addition, data were available for only three functional characteristics, whereas data were not available for soccer specific skills, for potentially relevant behavioral variables, and for coach perceptions of the players as youth. The limited sample sizes also did not permit comparisons of adult playing status by position.

## CONCLUSIONS

In summary, comparisons of the youth characteristics of those playing soccer as adults and those no longer involved in the sport indicated no consistent pattern of variation in growth and maturity status in each of the three competitive CA groups. considered. Among players 11–12 and 14–15 years, however, amateur and professional participants in soccer tended to perform better than those no longer partitipating in the sport in the standing long jump and sprint/agility tasks, though the three groups did not differ in the endurance tasks. Among players 16–17 years, on the other hand, no differences were noted among the three playing status groups for the three physical performance tasks. The proposed hypothesis that variation in the growth, maturity and functional characteristics of youth soccer players who persist in the sport into adulthood would emerge after the interval of the adolescent growth spurt was thus not supported by the available data. The results highlight the need to consider soccerspecific skills and coach perceptions of skills, and potential interactions with the growth, maturity, functional and perhaps behavioral characteristics of youth players. Nevertheless, results of the study may be of interest to coaches and others working with youth athletes and to sports scientists interested in the anthropometric and performance characteristics and maturity status of youth players and other factors that may contribute to successful careers in soccer.

### Author contributions

RMM, LH, JPD, MJCS: conceptualization, manuscript preparation; LH: data collection; JPD, TO, NSB, MJCS: database organization; RMM: x-ray film readings; RMM, LH, AS; MJCS: formal analysis, data modeling, statistics; MJCS, JPD, AS, JMCM, OMT: funding for data publication fee; RMM, LH, TO, JK, AS, MJCS: manuscript draft; RMM, LH, TO, NSB, OMT, MJCS: editing and submission preparation; RMM, LH, JK, MJCS: writing revised version; RMM, LH, JPD, TO, NSB, JMCM, OMT, AS, JK, MJCS: read and approved the final version.

## Data Availability

The data that upon which the study is based are not publicly available due to departmental policy and privacy commitments to the study participants. Nevertheless, the data may be available upon reasonable request to Professor Manuel J. Coelho-e-Silva, Faculty of Sports Science and Physical Education, University of Coimbra, Portugal.
